# Supplementary material for: Relationship between Physical Activity and Sedentary Behavior, Spinal Curvatures, Endurance and Balance of the Trunk Muscles-Extended Physical Health Analysis in Young Adults
Source: Int J Environ Res Public Health. 2023 Oct 18;20(20):6938. doi: 10.3390/ijerph20206938 (PMC10606682; doi:10.3390/ijerph20206938)
Supplement: Supplementary file 1 [file ijerph-20-06938-s001.zip › ijerph-2590477-supplementary.pdf]

International Journal of
Environmental Research and Public Health

Supplementary Materials

Relationship between Physical Activity and Sedentary Behavior, Spinal Curvatures, Endurance and Balance of the Trunk Muscles-Extended Physical Health Analysis in Young Adults

Verner Marijančić [1], Tanja Grubić Kezele [2,3,*], Stanislav Peharec [1], Nataša Dragaš-Zubalj [4], Sandra Pavičić Žeželj [5] and Gordana Starčević-Klasan [6]

1. Department of Physiotherapy, Faculty of Health Studies, University of Rijeka, 51000 Rijeka, Croatia; verner.marijancic@uniri.hr (V.M.); stanislav.peharec@fzsri.uniri.hr (S.P.)
2. Department of Physiology, Immunology and Pathophysiology, Faculty of Medicine, University of Rijeka, 51000 Rijeka, Croatia
3. Department of Clinical Microbiology, Clinical Hospital Rijeka, 51000 Rijeka, Croatia
4. Department of School and University Medicine, Teaching Institute of Public Health of Primorje-Gorski Kotar County, 51000 Rijeka, Croatia; natasa.dragas-zubalj@zzjzpgz.hr
5. Department of Health Ecology, Teaching Institute of Public Health of Primorje-Gorski Kotar County, 51000 Rijeka, Croatia; sandrapz@medri.uniri.hr
6. Department of Basic Medical Science, Faculty of Health Studies, University of Rijeka, 51000 Rijeka, Croatia; gordanask@fzsri.uniri.hr

* Correspondence: tanja.grubic@uniri.hr

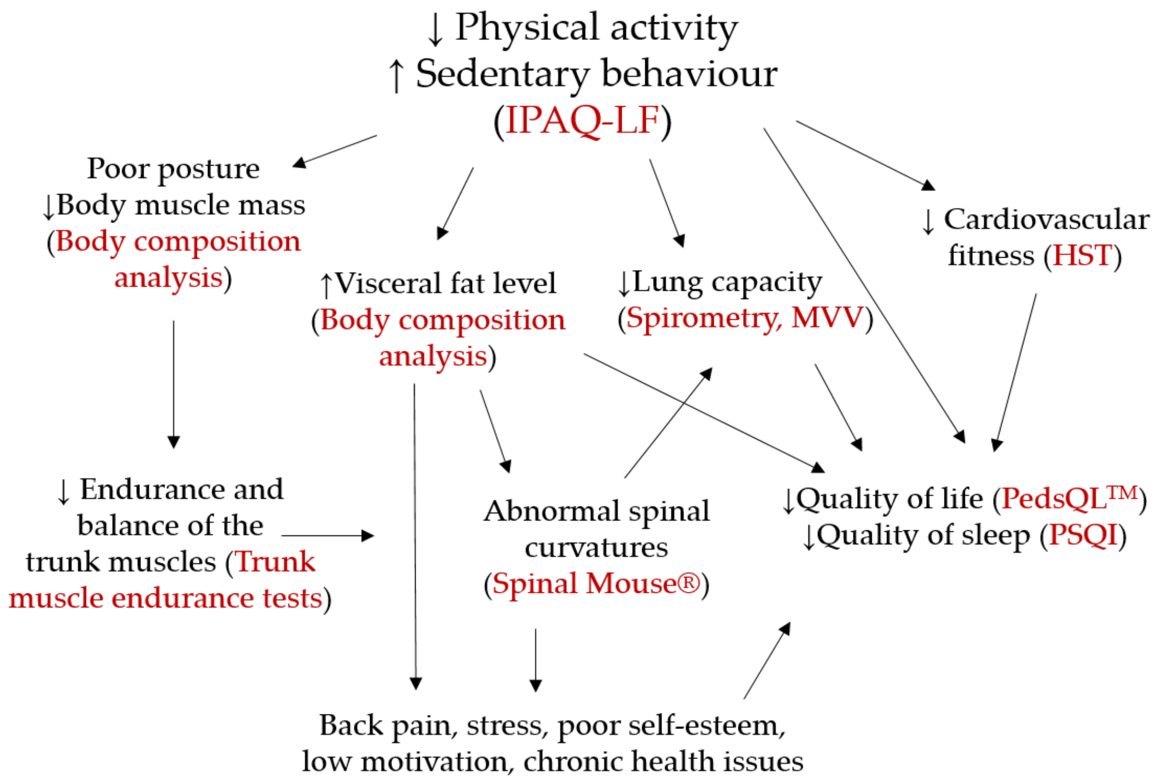

Figure S1. Interplay of possible consequences of low physical activity and sedentary behavior with accompanying measurement methods. IPAQ-LF, the International Physical Activity Questionnaire-Long Form; HST, Harvard step test; PedsQL™, Pediatric Quality of Life Inventory™; PSQI, Pittsburgh Sleep Quality Index; MVV, Maximal Voluntary Ventilation.

Table S1. Pearson correlation analysis for spinal curvatures vs. BMI, body composition, quality of life and sleep, endurance and balance of the trunk muscles, cardiovascular fitness and respiratory function.

**N = 82**		**Spinal curvatures**
**Variable**	Thoracic kyphosis angle - straight posture (°) r/p	Lumbar lordosis angle - straight posture (°) r/p
**BMI (kg/m²)**	0.026/0.849	-0.167/0.216
**Body composition**		
Visceral fat level	0.084/0.535	-0.209/0.121
Body fat (%)	-0.140/0.302	0.277/0.038*
Muscle mass (%)	0.143/0.293	-0.284/0.034*
**Quality of life (PedsQL™)**		
Physical health	0.191/0.159	-0.017/0.899
Psychosocial health	0.002/0.985	0.068/0.615
Total score	0.077/0.568	0.044/0.745
**Sleep quality (PSQI)**	-0.012/0.925	0.017/0.897
**Trunk muscle endurance**		
Trunk flexor test (sec)	-0.128/0.346	-0.142/0.297
Trunk extensor test (sec)	-0.132/0.331	0.264/0.049*
Trunk extensor/flexor test ratio	0.002/0.985	0.323/0.015*
**Cardiovascular fitness (HST)**	-0.180/0.182	-0.247/0.066
**MVV (L/min) (Spirometry)**	0.102/0.451	-0.289/0.030*

*statistical significance; BMI, Body Mass Index; N, number; r, Pearson correlation coefficient; p, level of statistical significance; PSQI, Pittsburgh Sleep Quality Index; PedsQL™, Pediatric Quality of Life Inventory™; HST, Harvard Step Test; MVV, Maximal Voluntary Ventilation.
